# The Voltage-Dependent Anion Channel 1 (AtVDAC1) Negatively Regulates Plant Cold Responses during Germination and Seedling Development in Arabidopsis and Interacts with Calcium Sensor CBL1

**DOI:** 10.3390/ijms14010701

**Published:** 2013-01-04

**Authors:** Zhi-Yong Li, Zhao-Shi Xu, Guang-Yuan He, Guang-Xiao Yang, Ming Chen, Lian-Cheng Li, Youzhi Ma

**Affiliations:** 1 The Genetic Engineering International Cooperation Base of Chinese Ministry of Science and Technology, Chinese National Center of Plant Gene Research (Wuhan) HUST Part, College of Life Science and Technology, Huazhong University of Science & Technology (HUST), Wuhan 430074, China; E-Mails: lizy83@yahoo.cn (Z.-Y.L.); hegy@hust.edu.cn (G.-Y.H.); ygx@mail.hust.edu.cn (G.-X.Y.); 2 Institute of Crop Science, Chinese Academy of Agricultural Sciences (CAAS)/National Key Facility for Crop Gene Resources and Genetic Improvement, Key Laboratory of Biology and Genetic Improvement of Triticeae Crops, Ministry of Agriculture, Beijing 100081, China; E-Mails: chenming@mail.caas.net.cn (M.C.); lilch@mail.caas.net.cn (L.-C.L.)

**Keywords:** Arabidopsis, voltage-dependent anion channel, cold stress, germination, calcium, interaction protein

## Abstract

The voltage-dependent anion channel (VDAC), a highly conserved major mitochondrial outer membrane protein, plays crucial roles in energy metabolism and metabolite transport. However, knowledge about the roles of the VDAC family in plants is limited. In this study, we investigated the expression pattern of *VDAC1* in Arabidopsis and found that cold stress promoted the accumulation of *VDAC1* transcripts in imbibed seeds and mature plants. Overexpression of *VDAC1* reduced tolerance to cold stress in Arabidopsis. Phenotype analysis of *VDAC1* T-DNA insertion mutant plants indicated that a *vdac1* mutant line had faster germination kinetics under cold treatment and showed enhanced tolerance to freezing. The yeast two-hybrid system revealed that VDAC1 interacts with CBL1, a calcium sensor in plants. Like the *vdac1*, a *cbl1* mutant also exhibited a higher seed germination rate. We conclude that both VDAC1 and CBL1 regulate cold stress responses during seed germination and plant development.

## 1. Introduction

The voltage-dependent anion channel (VDAC) encoded by a small gene family is the major transport protein in the outer membrane of mitochondria, which are present in all organisms from fungi to animals and plants. In *Saccharomyces cerevisiae*, there are two *VDAC* genes and mammals, including mice and human, have three isoforms [1,2]. Genomic sequence analysis revealed that there are five *VDAC* isoforms in Arabidopsis [3]. At least three isoforms are present in *Oryza sativa* (rice) and *Nicotiana tabacum* (tobacco) [4–6]. Five different *VDACs* have been identified in *Lotus japonicas* and *Medicago truncatula* [3,7]. Up to now, *VDAC* isoforms have been identified in maize, wheat and rape [8,9]. Plants appear to contain more *VDAC* isoforms than yeast and mammals.

*VDACs* are thought to function in the regulation of metabolite transport between mitochondria and the cytoplasm [10,11]. In mammals *VDAC*, also known as a component of the permeability transition pore (PTP) complex formed in the junction site of mitochondrial outer and inner membrane, is a key player in mitochondria-mediated cell death [12,13]. The general importance of *VDAC* in plant has only recently emerged. *VDAC* might be the important component of the tRNA import machinery in plant mitochondria [14–16]. Besides their role in metabolite transport, plant *VDACs* are also involved in programmed cell death [17]. The expression of *VDACs* in plants can be affected by different abiotic and biotic stresses, including cold, drought, salinity and pathogen defense. Differential expression of *VDAC* genes in response to such stresses has been reported. Four *VDAC* isoforms from Arabidopsis were upregulated in response to a bacterial pathogen, but were not affected by salt, cold or drought stresses during 24 h [18]. A pearl millet *VDAC*, identified as a salinity-inducible gene, was also upregulated by drought, cold and salicylic acid, but not by abscisic acid [19]. Three isoforms of tobacco VDACs were upregulated by inoculation with a non-host bacterial pathogen, but not by the host pathogen [6]. The above data indicate that the expression of plant *VDAC* members is often modulated by certain abiotic and biotic stimuli. Characterization of the biochemical and physiological functions of *VDAC* isoforms in plants have also been characterized. Three of the *VDACs* in Arabidopsis, viz. *VDAC1*, *VDAC2* and *VDAC4*, may play important roles in plant growth [20,21]. Transgenic Arabidopsis plants suppressing *AtVDAC2* expression showed an abscisic acid (ABA)-insensitive phenotype, suggesting the role of VDAC in ABA signaling [22]. Transgenic rice plants overexpressing millet VDAC became tolerant to salinity stress [19]. Thus the fine-tuned enhancement of *VDAC* expression may improve tolerance to environmental stresses.

Nevertheless, information regarding plant *VDACs* is fragmented, and individual functions and the physiological significance of *VDAC* isoforms are still needed to be well studied. This prompted further investigation of *VDAC* members from plant species. The VDAC family in Arabidopsis was selected here for examination, because Arabidopsis is a model plant. In this study, we found that *VDAC1* was involved in cold response during germination and plant development. By using the yeast two-hybrid system, a potential interactor of VDAC1 was identified as the unique plant Ca^2+^ sensor (calcineurin B-like) CBL1. The biological relevance of VDAC1 and CBL1 is further discussed.

## 2. Results

### 2.1. Expression Patterns of the *VDAC1* in Arabidopsis

**Figure 1:**
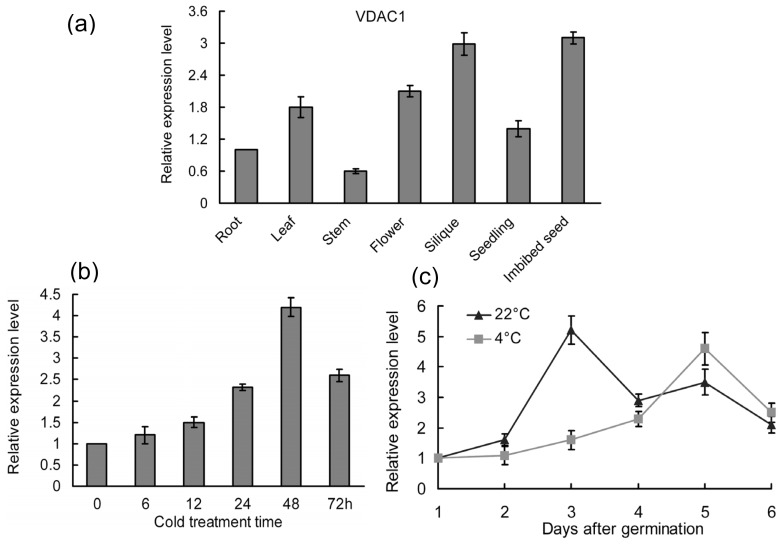
Expression patterns of *VDAC1* in Arabidopsis. (**a**) Expression of *VDAC1* in various tissues or organs. The transcript level in roots was used as the control, where the *VDAC1* mRNA level is given as 1. (**b**) Expression analysis of *VDAC1* under cold treatment. (**c**) Real-time PCR analyses of *VDAC1* expression in wild-type during seed germination under normal conditions (22 °C) or low temperature (4 °C). The expression of *VDAC1* was normalized to the expression of *UBQ10* in each PCR assay. Means and SD were calculated from three independent experiments.

To better understand the physiological function of *VDAC1* in Arabidopsis, we designed real-time PCR experiments to examine the genetic temporal and spatial expression changes in detail. As shown in Figure 1a, *VDAC1* transcripts were abundant in imbibed seeds and siliques. At*VDAC1* expression was previously shown to be not affected by several abiotic stresses [18]. However, *VDAC1* was promoted after 48 h under cold stress in mature plants in this study (Figure 1b). VDAC1 perhaps plays a role in cold response at the mature stage. In addition, *VDAC1* was up-regulated at either 22 or 4 °C during seed germination (Figure 1c). The mRNA level of *VDAC1* exhibited a sudden increase at 3 days or 5 days, when wild-type seeds were germinated at 22 or 4 °C, respectively, indicating *VDAC1* involvement in seed germination.

### 2.2. VDAC1 Overexpressing Arabidopsis Plants is Sensitive to Cold Stress

A comparative analysis was done to investigate the physiological roles of *VDAC1* on germination and response to cold stress. To examine germination and seedling growth of *VDAC1* overexpressing plants, a *vdac1* mutant, and wild-type (WT) (Columbia) were examined under designated conditions.

**Figure 2:**
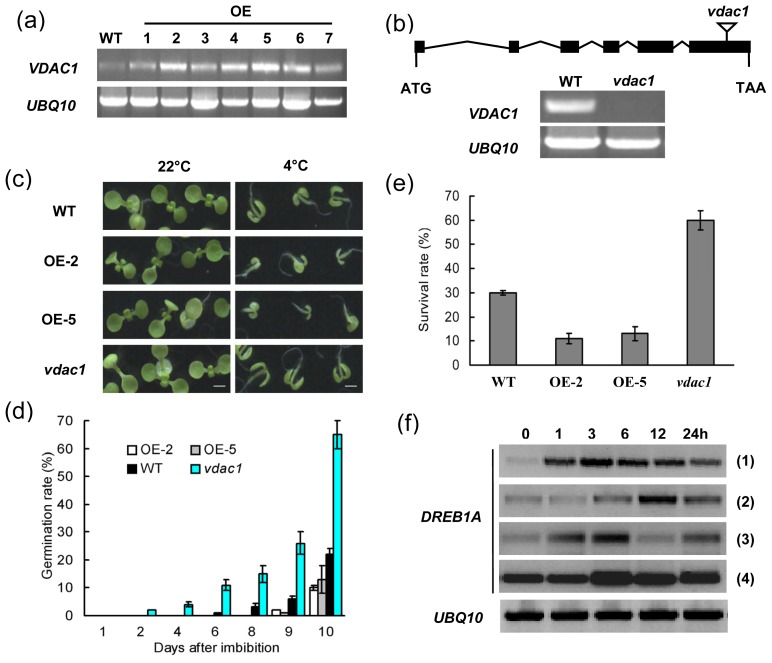
Involvement of *VDAC1* in seed germination and cold stress response. (**a**) RT-PCR expression analysis of *VDAC1* in different lines. Transcripts of *VDAC1* in wild-type (WT) and transgenic Arabidopsis lines (OE-1 to 7) were detected using semi-quantitative RT-PCR with 23 and 26 reaction cycles for *VDAC1* and *UBQ10*, respectively. (**b**) *VDAC1* gene structure and RT-PCR analysis of the WT and *vdac1* mutant. Introns are shown as lines, and exons are shown as black boxes. The triangle indicates the location of the T-DNA insertion site in *vdac1*. The numbers of semi-quantitative RT-PCR reaction cycles for *VDAC1* and *UBQ10* were 26. (**c**) Representative images of different phenotypes at day 15 after imbibition at (22 °C) or 4 °C. Bars = 2 mm. (**d**) Seed germination frequencies in WT, *vdac1* mutant and transgenic plants (OE-2 and OE-5) at 4 °C. Data points represent the mean ± SE of three independent biological determinations. (**e**) Survival rates after cold stress. Survival rates were calculated 48 h after re-watering. Data represent means ± SD (*n* = 20). (**f**) Semi-quantitative RT-PCR expression analysis of cold-responsive *DREB1A* gene with gene specific primers: 5′-TCTCTGAACCAGAGTCGTTT-3′ (forward) and 5′-CTTCTTCTCACCGTCTTCAC-3′ (reverse) in WT (1), transgenics (2 and 3) and *vdac1* mutant (4) plants induced by cold stress. Similar results were obtained in at least three biological replicates. The *UBQ10* gene was amplified as an internal control.

Semi-quantitative analysis showed that *VDAC1* mRNA levels in transgenic Arabidopsis lines were much higher level than in WT plants (Figure 2a). The *vdac1* mutant with a T-DNA insertion site in the last exon of the genome sequence had no detectable *VDAC1* transcript (Figure 2b). As shown in Figure 2c,d, there was no visible difference when seeds of the *vdac1* mutant, transgenics and WT were germinated on MS medium under normal conditions (22 °C). However, seeds of WT and *VDAC1* overexpressing lines (OE-2 and OE-5) germinated more slowly than the *vdac1* mutant at 4 °C, and the root growth and cotyledon expansion of transgenic seedlings were severely inhibited compared with either WT or mutant plants. The germination rates of the *VDAC1* overexpressing lines were even lower than the WT. These results demonstrated that VDAC1 functions during seed germination and seedling growth at low temperatures.

We also compared the survival rates of the *VDAC1* overexpressing lines with those of WT and *vdac1* mutant plants following cold stress. As shown in Figure 2e, the *VDAC1* overexpressing lines were less tolerant of the freezing treatment than WT plants, whereas with the same treatment, 60% of *vdac1* mutant plants survived compared with a 30% of WT plants. In addition, cold induction of *DREB1A* was less in the *VDAC1* overexpressing lines compared with WT control plants (Figure 2f). Moreover, expression of *DREB1A* was obviously enhanced in *vdac1* mutant plants relative to in WT. The results from the germination and freezing tolerance assays support the idea that VDAC1 functions cold stress response in plants.

### 2.3. Interaction of VDAC1-CBL1

**Figure 3:**
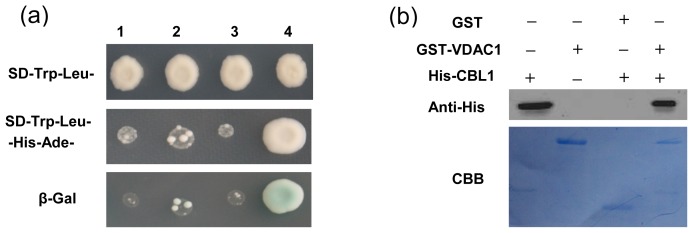
Interaction between VDAC1 and CBL1. (**a**) Yeast two-hybrid interactions. Vectors were co-introduced into yeast AH109 strain in different combinations: 1) pGADT7 and pGBKT7; 2) pGADT7-CBL1 and pGBKT7; 3) pGADT7 and pGBKT7-VDAC1; and 4) pGADT7-CBL1 and pGBKT7-VDAC1. Transformants were placed on the selection medium and grown for four days before the β-galactosidase (β-Gal) assay. (**b**) *In vitro* GST pull-down assay. GST-VDAC1 and His-CBL1 were expressed in *E. coli* and used for analysis. The presence or absence of each protein in the reaction mixture is shown as + or −, respectively. After transformed from the SDS-PAGE gel, the Polyvinylidene Fluoride (PVDF) membrane was stained with Coomassie Brilliant Blue (CBB). Unrelated His-tagged recombinant CBL1 protein at the left channel was used as a control. Experiments were performed three times, and a representative result is shown.

Interactions of proteins with other proteins or nucleic acids are important for most biological functions. A search for interacting partners is necessary to understand the functions of VDAC1. A positive interactor of VDAC1 was identified, and sequence analysis revealed that the VDAC1-interacting protein was CBL1. In yeast two-hybrid screening, strong growth on SD-Trp-Leu-Ade-His medium and activity of the reporter gene were observed only in yeast cells co-transformed with pGBKT7-VDAC1 and pGADT7-CBL1 (Figure 3a), indicating interaction of VDAC1 and CBL1 in yeast. His-CBL1 also physically interacted with GST-VDAC1, as shown by anti-His antibody on the resulting western blot (Figure 3b). These results demonstrated that VDAC1 interacts with CBL1.

### 2.4. cbl1 Was Insensitive to Low Temperature during Seed Germination

**Figure 4:**
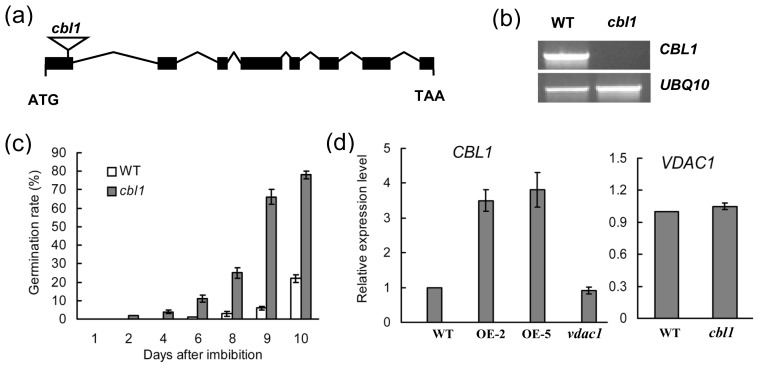
CBL1 is involved in seed germination at 4 °C. (**a**) The gene structure of *CBL1* and T-DNA insertion sits in the *cbl1* mutants. Introns are shown as lines and exons as black boxes. Triangle indicates the location of the T-DNA insertion site. (**b**) RT-PCR analysis of the WT and *cbl1* mutant. The *CBL1* transcript was absent in the *cbl1* mutant. The *UBQ10* transcript was amplified as an internal control. (**c**) Seed germination frequency in WT and *cbl1* mutant at 4 °C. Data points represent the mean ± SE of three independent biological determinations. (**d**) Relative expression levels of *VDAC1* and *CBL1* are quantified in *cbl1* and *VDAC1* overexpressing plants (OE-2 and OE-5). Mean values were normalized to the transcript level of the internal control, *UBQ10*.

Previous study showed that disruption of CBL1 promoted freezing stress tolerance in Arabidopsis [23]. Here, seed germination under cold treatment of a *CBL1* T-DNA insertion mutant was evaluated. The *cbl1* mutant had a T-DNA insertion site in the first exon of *CBL1* and did not produce a detectable *CBL1* transcript in a reverse transcription experiment (Figure 4a,b). There were no obvious morphological or developmental differences between WT and *cbl1* mutant plants grown on MS medium at 22 °C. However, seeds of WT germinated more slowly than the *cbl1* mutant on MS medium at 4 °C (Figure 4c). This suggests that *CBL1* mutation alters cold stress response during seed germination.

## 3. Discussion

Plant research to date has mainly focused on identification and expression patterns of VDAC isoforms. There is limited data on the functions of *VDAC* genes. Specific roles could be determined through *VDAC* knock-down or knock-out experiments. Previous studies revealed that *VDAC1* is associated with mitochondria, and its disruption affects pollen germination and pollen tube growth in Arabidopsis [20,21]. In this study, we gained insight into the novel roles of VDAC1 in Arabidopsis based on comparisons of overexpressing transgenic lines, wild-type and T-DNA insertion mutants.

The time, location and levels of gene transcripts are finely regulated in all organisms, ensuring normal development and optimal survival of plants under both normal or stress conditions. Spatiotemporal expression profiles of *VDAC1* in Arabidopsis were described in transgenic Arabidopsis plants carrying *VDAC1* promoter-GUS constructs [20]. The result showed that the *VDAC1* promoter was active in shoot meristems and surrounding tissues. Here, *VDAC1* transcripts were detected in all tested tissues and organs, which is consistent with the analysis of the microarray expression database [24]. However, it was strongly expressed in flowers and siliques (Figure 1a); not exactly the same as in the previous study, which showed the level of transcripts of *VDAC1* decreases with the stage of development in Arabidopsis [11]. More research is needed to elucidate this significant difference. In addition, *VDAC1* was obviously upregulated during seed germination (Figure 1c), which agrees with the data given by the Genvestigator microarray expression database [24], hence indicating an important role in seed germination. Previous studies showed that down-regulated VDAC expression, or expression based on mutants, could lead to changes in channel activities, such as cytosolic ATP levels and mitochondrial ATP-synthesis rates [25–27]. For instance, reduction in the expression of *VDAC1* in human cells led to a decrease in the ATP level, suggesting a demand on VDAC proteins for control of ATP flux [26]. Plant VDAC proteins may share similar functions in control of ATP levels, which are important for degradative metabolism. Germination of seeds following a dormant stage requires an efficient energy source that comes from degradative metabolism [28]. Thus, the high *VDAC1* gene expression level observed in imbibed seeds could be explained by the energy demand for seed germination. Sequent study showed that *VDAC1* overproduction increased cold stress sensitivity during both seed germination and seedling growth, whereas *vdac1* T-DNA insertion lines exhibited a higher seed germination frequency than *VDAC1* overexpressing and wild-type lines at 4 °C (Figure 2d). In addition, a loss-of-function mutant *vdac1* in Arabidopsis showed enhanced tolerance to freezing (Figure 2e). These results suggest that VDAC1 plays a negative role in cold stress response.

In animal cells, several proteins were suggested to interact with VDAC [29]. These included mitochondrial creatinekinase, microtubule-associated protein 2, mitochondrial heat shock protein 70 and several glycolytic enzymes, such as hexokinase (Hxk), aldolase, and glyceraldehyde 3-phosphate dehydrogenase (GAPDH). In Arabidopsis plants, Hexokinase 1 (HXK1) is an interaction protein of VDAC, and *AtHXK1* overexpressing transgenic plants have faster germination kinetics, which coincides with observations on seed germination of AtVDAC2 antisense transgenic lines in the presence of ABA [22,30]. To gain insights into how VDAC1 affects plant response to cold stress, we performed a yeast two-hybrid screen using VDAC1 as bait. A few polypeptides were identified as potential interaction partners, among which we focused on the Ca^2+^ sensor, CBL1.

In Arabidopsis, *CBL1* is activated by various abiotic stresses and functions as a positive regulator in salt or drought stress responses [23]. It was also described as a negative regulator of cold response [23]. By contrast, *VDAC1* was stable under salt, cold or drought stresses in Arabidopsis during 24 h detected by semi-quantitative RT-PCR [18], whereas its expression was enhanced in mature plants after 48 h under cold stress examined by real-time PCR method (Figure 1b), indicating a possible role in cold stress response. Overexpression of *VDAC1* reduced freezing tolerance, whereas *vdac1* mutant plants showed superior performance to WT under freezing stress, suggesting that VDAC1 functions as a negative regulator in cold stress response. This is consistent with the role of CBL1 in cold stress response. CBL1 was shown to alter the expression of a series of cold-induced genes when subjected to cold stress [23]. To test if VDAC1 and CBL1 could be involved in the regulation of a common set of genes, the expression of genes found to be altered by CBL1 under cold treatment were quantified in *VDAC1* overexpressing and *vdac1* mutant lines. Among these genes, the expression of *DREB1A*, a gene specifically responsive to cold, was inhibited in *VDAC1*-overexpressing plants, suggesting that *VDAC1* overexpression exerted negative regulation on *DREB1A*. On the contrary, *DREB1A* was highly induced in *vdac1* mutant plants (Figure 2f). These results indicate that VDAC1 and CBL1 have similar effects on the expression of *DREB1A* [23]. The *vdac1* mutant also showed superior performance during seed germination under low temperature, and the phenotypes observed for *cbl1* resembled those of *vdac1* (Figure 4c). To determine if the visual phenotypes of the mutants at 4 °C were due to quicker germination, but not faster growth, seeds were germinated at 22 °C for two days before being transferred to 4 °C. The mutants did not present any obvious morphological differences (data not shown). These results demonstrated that both VDAC1 and CBL1 function during seed germination at low temperature, suggesting a biological relevance of the two genes.

To analyze a possible cross-regulation between VDAC1 and CBL1, their mRNA levels in *cbl1* and *VDAC1* overexpressing plants were quantified. As shown in Figure 4d, no significant differences in mRNA levels of *VDAC1* were detected between the wild-type and *cbl1* mutant plants. The mRNA level of CBL1 was also found to be stable in *vdac1* mutant plants. However, *CBL1* was induced more than three-fold in *VDAC1* overexpressing plants. Previous studies showed that Ca^2+^ flux across the mitochondria outer membrane occurs through VDAC and VDAC has a role in regulation of the Ca^2+^ homoeostasis [31]. Perhaps, overproduction of *VDAC1* promotes calcium flux from mitochondria and then triggers expression of *CBL1*, which functions as a unique Ca^2+^ sensor in plants [32]. VDAC proteins could regulate Ca^2+^ permeation to sense changes in the physiological state of the cell [31,33]. Deletion of *VDAC1* may modify the perception of calcium influx by cold stress to result in altered cold-induced signaling and adaptation. However, whether and how VDAC proteins influence calcium flux in plant cells remain to be elucidated.

It was found that the VDAC (At5g15090) protein was improved during heat- and senescence-associated cell death, but its corresponding transcripts did not show any change [34,35]. And, three rice *VDACs* (OsVDAC1-3) were found to be up-regulated in roots during the recovery period from osmotic stress, whereas the overall VDAC protein levels stayed constant [5]. There might be no obvious linear relationship between the level of transcripts and that of the corresponding active protein products, which are the functional elements of cells, tissues and organisms [11]. These data suggest that there is a post-transcriptional regulation in VDAC protein level. In this study, expression of *VDAC1* was promoted in normal conditions (22 °C) during germination. However, it seems that VDAC1 is not essential for germination in normal conditions (22 °C), as shown in Figure 2. In addition, VDAC1 were found to be promoted slowly, which displays some inconsistencies with the previous results. It perhaps is due to the post-transcriptional regulation of *VDAC1* or other unexpected mechanisms. More research is needed to answer such questions.

## 4. Experimental Section

### 4.1. Plant Material and Growth Conditions

Seeds of Arabidopsis (Columbia strain), mutant lines *vdac1* (SALK_034648) and *cbl1* (SALK_110426) were surface sterilized with bleach and thoroughly washed five times with sterile water before incubation in a growth chamber following three days of cold treatment. For the mutant screen, seedlings were transferred from plates to soil and grown at 22 °C in a growth room with a 16 h photoperiod. All plant materials were harvested and stored at −80 °C for DNA and RNA isolation.

### 4.2. Screening of T-DNA Insertion Mutants

Plant DNA isolation was performed as described [36]. The locations of the T-DNA insertion sites in the *vdac1* and *cbl1* homozygous mutants were determined by direct sequencing of PCR products amplified using DNA as a template by the T-DNA border primer LBb1.3 [37] for SALK lines and gene-specific primers as follows: SALK_034648LP: 5′-TTATTACAGGCCAACAATGCC-3′ and SALK_034648RP: 5′-GTGATTGGCTCCAATGTCTTG-3′ for *vdac1*; SALK_110426LP: 5′-GGGCTACGATACATTGAATCG-3′ and SALK_110426RP: 5′-TTGATCGTCTGGTTTCGAATC-3′ for *cbl1*.

Homozygous mutant plants were further confirmed by RT-PCR with gene-specific primers of *VDAC1* (GenBank accession number: AT3G01280): 5′-CTTGCCCTTGGTACTGATGTTT-3′ (forward) and 5′-GAGGGTGTTCTCATTGGTTGAG-3′ (reverse) and of *CBL1* (GenBank accession number: AT4G17615): 5′-AATGAAACTGGCTGATGAAACC-3′ (forward) and 5′-CCTCCGAATGGAAGACAAAACT-3′ (reverse).

### 4.3. RNA Isolation and Real Time PCR

Total RNA was isolated from different plant materials using Trizol reagent (Takara, city, country), and reverse transcription were performed with 2 μg of total RNA for the first strand cDNA synthesis with a PrimeScript 1st Strand cDNA Synthesis kit (Takara, Dalian, China), according to the manufacturer’s instructions. Real-time PCR was performed with the following gene-specific primers: *VDAC1*, 5′-CAGAAGCTACGAGACCAGAAGTTG-3′ (forward) and 5′-CTATGGTACCATGAACCTTGCATG-3′ (reverse); and *CBL1*, 5′-TTTTTCCAGAGGCAATCATG-3′ (forward) and 5′-GCCCATTTGGTGGTATCTTC-3′ (reverse). Real-time PCR analyses were performed on an ABI7300 system with SYBR Premix Ex Taq II (Takara, Dalian, China), as previously described [38]. Relative quantitative results were calculated by normalization to *UBQ10* (GenBank accession number: AT4G05320).

### 4.4. Construction of Expression Vector and Isolation of Transgenic Plants

The open reading frame of *VDAC1* was amplified by PCR from the cDNA with the specific primers: 5′-AACCATGAGCAAAGGTCCAGGACTC-3′ (forward) and 5′-CTCAAGGTTTGAGAGAAGAGAGAGACC-3′ (reverse). PCR fragments were cloned into the pMD18-T vector (Takara, Dalian, China), and the resulting plasmid was sequenced. To construct the eukaryotic expression vector, the *VDAC1* fragment was cloned into the pBI121 previously digested with *Bam*HI and *Sac*I. After sequencing, the transgenic Arabidopsis (Columbia accession) lines were obtained by Agrobacterium-mediated dip flora using 50 mg/L kanamycin as a selective agent as, previously described [39].

### 4.5. Seed Germination Assay

For seed germination assays, seeds from the different genotypes were harvested from plants grown simultaneously in a glasshouse and then stored for four weeks. For the seed germination assay at 4 °C, about 80 sterile seeds for each genotype were kept in an incubator at 4 °C with a 16 h photoperiod. Germination was scored by radicle emergence. For the cold tolerance assay, four-week-old plants were treated at 4 °C for two days and then −6 °C for 2 h before recovery under normal growing condition. Survival rates were determined after re-watering for two days.

### 4.6. Yeast Two-Hybrid Screening

An Arabidopsis seedling cDNA library was constructed in a pGADT7-Rec2 vector containing a GAL4 activation domain using Matchmaker Library Construction (Clontech) and then transformed into yeast strain AH109 (Clontech). For yeast two-hybrid assays, the PCR product of *VDAC1* was cloned into the *Bam*HI and *Sal*I restriction sites of pGBKT7 vector (Clontech) to generate the pGBKT-VDAC1 bait vector. Screening of the cDNA library for candidate interaction partners of VDAC1 was performed using the MATCHMAKER two-hybrid system (Clontech). Transformants were selected by growth on a synthetic dropout (SD) medium that lacked Trp, Leu, Ade and His (SD-Trp-Leu-Ade-His), but supplemented with an optimal concentration of 3-amino-1,2,4-triazole (3-AT) to reduce any artificial interaction. Surviving clones were retransferred to SD-Trp-Leu-His-Ade-medium and assayed for β-galactosidase activity according to the manufacturer’s instructions (Clontech). For the yeast two-hybrid assay, the full coding sequence of *CBL1* was amplified with the specific primers: 5′-CGTCGCCGCACTCACTTT-3′ (forward) and 5′-TCCCACCAATCACATAACC TAAA-3′ (reverse) and cloned into the *Bam*HI and *Sac*I restriction sites of the pGADT7-Rec2 vector.

### 4.7. GST Pull-Down Assay

For the GST pull-down assay, the full coding fragment of Arabidopsis *VDAC1* was cloned into the *Bam*HI and *Sal*I restriction sites of the pGEX-4T-1 vector (Promega) to generate GST-VDAC1. The full length *CBL1* was cloned into the *Bam*HI and *Sac*I restriction sites of the pET28a (+) vector (Novagen) to generate His-CBL1. The recombinant proteins GST-VDAC1 and His-CBL1 were expressed in *Escherichia coli* strain BL21 (DE3) by induction with 0.5 mM isopropyl-1-thio-b-d-thiogalactoside (IPTG) and purified with glutathione sepharose or Ni-activated His-binding resin (GE Healthcare) according to the manufacturers’ instructions. Aliquots of GST and GST-VDAC1 beads (100 mL beads containing 15 mg of protein) were incubated overnight at 4 °C with the purified His-CBL1 protein on a rotary incubator. After being washed with ice-cold phosphate-buffered saline for five times, they were re-suspended in SDS gel-loading buffer and then loaded on SDS-PAGE (12%, *w*/*v*) gels. Protein bound to GST-VDAC1 was detected by western blotting using an anti-His antibody and visualized using chemiluminescence following the manufacturer’s recommendations (Amersham plc., Amersham, UK).

## 5. Conclusions

In this study, we investigated the expression pattern of VDAC1 in Arabidopsis. The results showed that *VDAC1* was redundant in imbibed seeds and induced by cold stress in mature plants. Further phenotype analysis confirmed that VDAC1 is involved in cold stress response during the seed germination and plant development. Using yeast two-hybrid system, a novel interactor of VDAC1 was identified as plant Ca^2+^ sensor CBL1. In summary, new insights were gained into the possible role of VDAC1 in Arabidopsis, particularly in showing a diversity of roles of the VDAC family in plants.
